# Hybrid nanocomposite curcumin-capped gold nanoparticle-reduced graphene oxide: Anti-oxidant potency and selective cancer cytotoxicity

**DOI:** 10.1371/journal.pone.0216725

**Published:** 2019-05-14

**Authors:** Lina A. Al-Ani, Wageeh A. Yehye, Farkaad A. Kadir, Najihah M. Hashim, Mohammed A. AlSaadi, Nurhidayatullaili M. Julkapli, Vincent K. S. Hsiao

**Affiliations:** 1 Institute of Postgraduate Studies, Nanotechnology & Catalysis Research Centre (NANOCAT), University of Malaya, Kuala Lumpur, Malaysia; 2 Department of Anatomy and Medical Imaging, Faculty of Medical and Health Sciences, University of Auckland, Auckland, New Zealand; 3 Department of Pharmacy, Faculty of Medicine, University of Malaya, Kuala Lumpur, Malaysia; 4 Centre for Natural Products and Drug Discovery (CENAR), University of Malaya, Kuala Lumpur, Malaysia; 5 University of Malaya Centre for Ionic Liquids (UMCiL), University of Malaya, Kuala Lumpur, Malaysia; 6 National Chair of Materials Sciences and Metallurgy, University of Nizwa, Nizwa, Sultanate of Oman; 7 Department of Applied Materials and Optoelectronic Engineering, National Chi Nan University, Nantou, Taiwan; Institute of Materials Science, GERMANY

## Abstract

Nanotechnology-based antioxidants and therapeutic agents are believed to be the next generation tools to face the ever-increasing cancer mortality rates. Graphene stands as a preferred nano-therapeutic template, due to the advanced properties and cellular interaction mechanisms. Nevertheless, majority of graphene-based composites suffer from hindered development as efficient cancer therapeutics. Recent nano-toxicology reviews and recommendations emphasize on the preliminary synthetic stages as a crucial element in driving successful applications results. In this study, we present an integrated, green, one-pot hybridization of target-suited raw materials into curcumin-capped gold nanoparticle-conjugated reduced graphene oxide (CAG) nanocomposite, as a prominent anti-oxidant and anti-cancer agent. Distinct from previous studies, the beneficial attributes of curcumin are employed to their fullest extent, such that they perform dual roles of being a natural reducing agent and possessing antioxidant anti-cancer functional moiety. The proposed novel green synthesis approach secured an enhanced structure with dispersed homogenous AuNPs (15.62 ± 4.04 nm) anchored on reduced graphene oxide (rGO) sheets, as evidenced by transmission electron microscopy, surpassing other traditional chemical reductants. On the other hand, safe, non-toxic CAG elevates biological activity and supports biocompatibility. Free radical DPPH inhibition assay revealed CAG antioxidant potential with IC_50_ (324.1 ± 1.8%) value reduced by half compared to that of traditional citrate-rGO-AuNP nanocomposite (612.1 ± 10.1%), which confirms the amplified multi-potent antioxidant activity. Human colon cancer cell lines (HT-29 and SW-948) showed concentration- and time-dependent cytotoxicity for CAG, as determined by optical microscopy images and WST-8 assay, with relatively low IC_50_ values (~100 μg/ml), while preserving biocompatibility towards normal human colon (CCD-841) and liver cells (WRL-68), with high selectivity indices (≥ 2.0) at all tested time points. Collectively, our results demonstrate effective green synthesis of CAG nanocomposite, free of additional stabilizing agents, and its bioactivity as an antioxidant and selective anti-colon cancer agent.

## Introduction

Among the various nanomaterials reported to date, graphene has ascended as the ‘wonder material’ and ‘shining star’, with a Nobel Prize awarded in 2010 for its discovery [1]. Due to the interesting chemical, mechanical, and optical properties, graphene applications have been actively pursued in the general biomedical fields, with distinctive focus on cancer nanomedicine, to confront the increasing cancer mortality rates [2–7]. In this context, graphene offers a range of nano-therapeutic modalities of phototherapy, drug delivery, and combination therapy [8–10]. This enables efficient support for functionalization and drug loading [11, 12], in addition to enhanced mechanical stability and cellular interactions [13]. The few limitations of graphene such as hydrophobicity and self-aggregation, can be effectively resolved via suitable functionalization and composite hybrid fabrication [14]. In fact, various studies have proven advantageous attributes using graphene-based composites (GBCs) in cancer nano-therapy, over raw un-functionalized graphene counterparts [15–17]. Nevertheless, the progress and development of these composites to the next *in vivo* and clinical stages remains slow [12]. The main barrier recognized is the safety profile, or biocompatibility and selectivity, of these composites towards normal tissue [12, 13]. After all, drug selectivity and enhanced patients’ life quality are the ultimate goal of nanotechnology in cancer treatment [18, 19].

For successful and selective cancer nano-therapy applications, more attention needs to be directed to GBCs fabrication and pre-clinical trials, as these processes constitute the basic foundation for further development and progress. In fact, careful design and suitable choice of raw materials and synthesis methodology are suggested approaches to increase GBCs biocompatibility [13, 20]. Accurate reporting of the selectivity index (SI) in pre-clinical *in vitro* testing is also an important factor determining safety and indicating successful employment of raw materials in cancer-active, normal cell-biocompatible composites. As such, current nanomedicine research indicates that graphene selection as a starting build-up template secures essential planar surface area that is suitable for functionalization and drug upload [21]. On the other hand, gold nanoparticles (AuNPs) stand out as a prominent functional moiety used heavily with various nanomaterials [11, 22], due to the stable [23], well-established biocompatible features that enhance the overall composite selectivity [11, 24]. Consequently, graphene-AuNPs hybrid composite is one of the best-studied materials in the field of cancer nano-therapy [25–29]. Furthermore, there is a mounting focus devoted to the hybridization and synthesis methodology used to combine different moieties into one nanocomposite. Green synthesis is recently identified as an easy, eco-friendly, cost-effective, safe, and efficient alternative to conventional chemical synthesis, further reinforcing biological activities of the whole system [30, 31]. As a result, there are various reports of GBCs synthesized by green methods (S1 Table), yet the majority show inconsistent use of human cancer/normal cell lines, with a lack of SI reporting in respective target tissues, which may contribute to indeterminate conclusions and hinder further development [13].

Apart from synthesis approaches, the suitable anti-cancer drug incorporation holds fundamental values and merits. Plant-derived drugs account for over 60% of drugs introduced worldwide recently [32], including *Curcuma longa* polyphenolic extract curcumin (CR), which has been the focal point of modern cancer research [33, 34]. Its major advantage lies in its multi-targeted therapeutic approach, which is of utmost importance to confer cancer disease that holds more than 500 gene products dysregulation [34, 35]. The astonishing ability of CR to inhibit tumor proliferation, cell cycle, metastasis, and angiogenesis is limited by its low bioavailability [34, 35]. Therefore, CR nano-formulations are prescribed as a valid solution [36]. In this study, we suggest, for the first time, the one-pot combination of all these beneficial attributes of graphene, AuNPs, and CR into one novel green nanocomposite, namely CR-capped AuNPs-reduced graphene oxide (CAG). Unlike previous reports of CR combining with GBCs through two-step chemical reactions [37, 38], this work proposes a novel green, simple, one-pot synthesis which uses CR to its fullest potential as a natural reducing agent, with capping and functionalization moiety during synthesis, while also preserving its anti-oxidant, cancer cytotoxic, and selective biological activities. This structure is anticipated to confer superior features, overcoming the limitations faced by each single component, in addition to being a green and safe product with no toxic impurities and increased biocompatibility. Comprehensive *in vitro* cancer cytotoxicity and respective selectivity indices are discussed in the current work, in terms of various concentrations, time points, and cell lines chosen to represent suitable target colon cancer tissue and distant normal liver cells, which mimic the probable *in vivo* behavior of GBCs as reported previously [39, 40]. We believe, as recommended by nano-toxicology reviews [41], that careful design and accurate SI measurement will improve knowledge gathered at the preliminary *in vitro* stage, creating a worthwhile template eligible for various biomedical applications and further study and development.

## Materials and methods

### Nanocomposite synthesis

#### GO synthesis

Graphite oxide was prepared using the ‘improved Hummer’s method’, as reported previously [42]. In brief, 1.0 g of graphite powder (Sigma Aldrich, < 20 μm) was mixed with 135 mL of 9:1 mixture of sulfuric to phosphoric acid, followed by slow addition of 6.0 g of potassium permanganate. The reaction mixture was then heated to 50°C and maintained in stirring conditions for 12 hours. The mixture was brought to room temperature and poured on iced deionized (DI)-water. In order to stop the reaction, dropwise addition of 30% hydrogen peroxide was performed, and a color change to yellow was observed. The yellowish suspension was then processed by multiple centrifugation and washing steps with DI-water, 30% HCl, ethanol, and DI-water again. The final pellet was dialyzed against DI-water using a dialysis membrane for 48 hours, and freeze dried.

#### CAG nanocomposite synthesis

The prepared graphite oxide powder was dispersed in DI-water and sonicated for 30 minutes in a bath sonicator to exfoliate sheets into graphene oxide (GO). The solution was then added to HAuCl_4_ solution (Sigma Aldrich), and aged for 30 minutes to promote gold ion attachment to the GO surface [43]. The mixture was then heated at 80°C, before CR solution was added (Merck, Germany). CR was first dissolved in DMSO, used as a stock solution, and then diluted with DI-water to give a 1 mM working solution. The pH of CR was adjusted (~9.0) with a weak base (sodium bicarbonate) prior to addition. After mixing all solutions in the flask, the reaction was kept at 80°C for 4 hours under stirring conditions. Later, the solution was brought to room temperature, and wash-spinned with DI-water several times to remove excess gold ions and control pH at ~7.0. The CAG pellet was finally obtained via freeze drying.

For comparison purposes, the rGO-AuNPs nanocomposite was synthesized by the traditional sodium citrate reduction method [43], and citrate-AuNPs were synthesized as described previously [44].

### Morphological and structural characterization

The synthesized materials were morphologically characterized by high resolution transmission electron microscopy (HR-TEM, Hitachi-HT7700, 120kv, Japan) to investigate the sheet-like morphology of rGO, and the formation of AuNPs and their size and dispersion patterns. A few drops of each sample were allowed to air-dry on a lacy copper grid coated with carbon film for TEM analysis. Graphene structural features were revealed using Raman spectroscopy (Renishaw inVia Raman, Gloucestershire, UK) with a 514 nm argon gas laser. Fourier-transform infrared spectroscopy (FT-IR) analysis was performed to detect functionalization, wherein the sample powder was ground with potassium bromide (KBr) into thin pellets. FT-IR spectra were recorded using a Spectrum 400 (PerkinElmer, Boston, MA, USA). X-ray diffraction patterns (XRD) were recorded using an EMPYREAN diffraction system with X-ray wavelength of 1.54060 Å. UV-Vis spectroscopic measurements were performed using PerkinElmer–Lambda 35 spectrophotometer at a wavelength range of 200–800 nm. Thermo-gravimetric analysis (TGA) was performed using a TGA/SDTA 851 (Mettler Toledo, USA) system with a heating rate of 10°C/min up to 800°C in a nitrogen environment. Dynamic light scattering (DLS) of CAG nanocomposite in cell culture RPMI-1640 medium was analyzed using Zetasizer Nano-ZS apparatus (Malvern Instruments, UK) at 25°C.

### Phenol content and anti-oxidant activity

To explore the presence of possible bioactive phenol moieties and the anti-oxidant activity of the CAG nanocomposite, total phenolic content (TPC) and DPPH (2,2-diphenyl-1-picrylhydrazyl) anti-oxidant tests were performed. TPC was measured using improved Folin-Ciocalteu (FC) protocol as described previously [45]. Briefly, 50 μL FC reagent (1:5 (v/v)) was mixed with 50 μL of sample in a 96-well plate (Nest Biotechnology, China). NaOH (0.35 M; 100 μL) was then added to the mixture and incubated for 3–4 minutes in the dark. Results were read at 760 nm absorbance of a microplate reader (Infinite-200Pro-TECAN). Ethanolic CR solution was used to create the standard calibration curve [46].

The anti-oxidant activity of the composite was evaluated using optimized DPPH assay [47, 48]. Ascorbic acid was used as a positive control in this experiment, while methanol served as a blank. Methanolic DPPH stock solution (2,2-diphenyl-1-picrylhydrazyl, Sigma-Aldrich) was prepared to yield a final concentration of 70 μM upon addition to sample (50–750 μg/mL). DPPH was added to all control and test tubes, followed by successive sonication (3 times, 3 minutes each, in dark) during 30 minutes of incubation. Absorbance was read at 517 nm using a microplate reader (Infinite-200Pro-TECAN). The percentage inhibition was calculated using Eq (1), where Abs. stands for absorbance measured at 517 nm:

%inhibition=(Abs.Control−Abs.Sample)/Abs.Controlx100 (1)

To determine IC_50_ values (sample concentration required to achieve 50% inhibition) of the DPPH radical, the percentage inhibition for each compound was plotted against different concentrations.

### Cell-based assays

#### Cell culture

Human colon cell lines HT-29 (colon adenocarcinoma), SW948 (Duke’s C colorectal carcinoma) and CCD841-Con (normal colon cells), in addition to human liver WRL-68 (normal hepatic cells) were all obtained from American Type Culture Collection (ATCC, Manassas, USA). Cells were cultured in RPMI-1640 medium (Sigma Aldrich, USA), supplemented with 10% fetal bovine serum (Gibco, USA), and 1% Pen-Strip antibiotic (10,000 units penicillin-10 mg streptomycin/mL, Sigma Aldrich, USA) in a 37°C, 5% CO_2_ incubator (Thermo Fisher Scientific, USA).

#### Cell Morphology using optical microscopy imaging

Cancer cells’ morphological changes upon nanocomposite exposure were observed using phase-contrast optical microscopy. Cells were plated in 24-wells plate (5 x 10^4^ cells/ml) and incubated overnight for attachment. Culture media was then removed and replaced with CAG suspension in media at low concentration (62.5 μg/ml) and high concentration (250 μg/ml) for 24 hours. Untreated cells were used as control, while treatment with raw material GO and traditional synthesized nanocomposite rGO-AuNPs were used for comparison. Phase-contrast bright field images were recorded via inverted microscope Eclipse Ti-S (NIKON Instruments, USA).

#### Cellular viability assay using WST-8

Cellular viability was assessed in CAG-treated cancer and normal cells using water soluble tetrazolium test (WST-8). Cell counting kit-8 (CCK-8, Sigma Aldrich, USA) was applied following the manufacturer’s protocol. Typically, 5000 cells were seeded in 96-well plates and incubated overnight for attachment. Following that, the medium was removed and cells were treated with 100 μL of different concentrations of CAG suspensions in media. After each time point (24, 48, and 72 hours), 10 μL of CCK-8 solution was added to each well, and incubated for a further 4 hours at 37°C, 5% CO_2_. At the end of incubation period, 80 μL was transferred from each well to a new 96-well plate, to avoid residual CAG precipitate that may affect absorbance readings [49]. The test was conducted in triplicate. Results were recorded using 450 nm absorbance value by (Infinite-M200Pro-TECAN). The percentage cell viability was calculated according to Eq (2):

Cellviability(%)=SampleAbsorbance/ControlAbsorbancex100 (2)

For comparison purposes, WST-8 test was performed as well using GO and rGO-AuNPs following the same assay conditions.

#### Selectivity index (SI)

The degree of selectivity of composite to the cancer cell line is explored as SI ratio as per previous reports [50, 51], according to the following Eq (3):

SI=IC50compositeonnormalcellline/IC50compositeoncancercellline (3)

#### Statistical analysis

Numerical results were expressed as the mean ± standard error of the mean (SEM) from triplicate experiments. Statistical significance was determined using factorial analysis of variance (ANOVA) test, with p value set at < 0.05 as accepted level of significance. All analyses were performed using SPSS software (Statistical Packages for Social Science, version 22.0, IBM Corporation, NY, USA).

## Results and discussion

### Integrated hybridization with multi-functional approach

**Fig 1:**
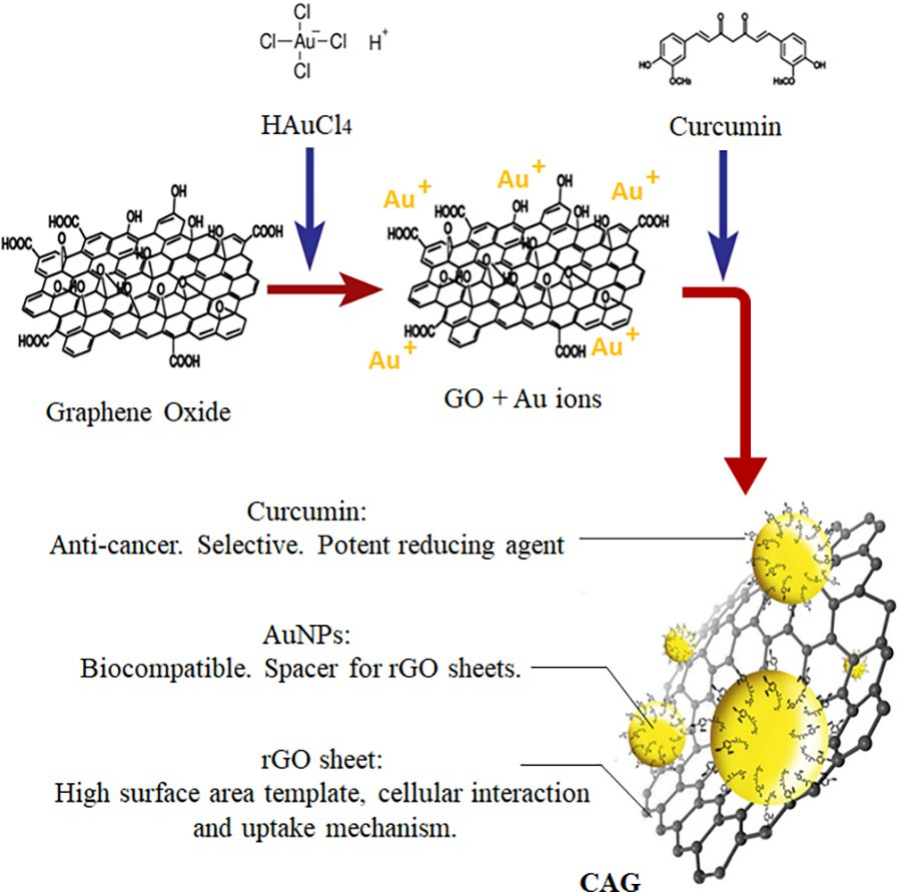
CAG nanocomposite synthesis illustration.

One of the promising concepts behind nanotechnology is hybridization, the remarkable outcome of which is a composite that merges various moieties, producing superior features with enhanced performance. For that reason, a novel, hybrid, and green incorporation of valuable well-studied nanomaterials of graphene, AuNPs, and natural CR into one nanocomposite is proposed as a multi-functional biocompatible agent, turning previous limitations into advantages. As illustrated in Fig 1, graphene in a CAG structure works as a template with a high planar surface area for maximal upload of AuNPs and the anti-cancer drug CR [11, 12]. The lipophilic nature of graphene is of major significance, ensuring cellular uptake and thus delivery of treatment cargo into cancer cells, surpassing membrane-penetration obstacles [12, 52], and low CR bioavailability and absorption deficiency [53]. On the other hand, AuNPs as a functional tool acts as a spacer preventing graphene sheet aggregation, which would adversely affect cellular viability and colloidal stability attributes [54]. The biocompatible nature of core gold also adds to the selectivity profile of the whole system [55, 56]. Curcumin outstandingly serves dual roles in the synthesized CAG nanocomposite as an anti-cancer drug and a potent green reducing agent for simultaneous gold salt and GO reduction and composite fabrication. This synthesis approach skipped the conventional use of chemical stabilizers or capping agents needed for stable unaggregated AuNPs formation [43, 57], thus providing a novel green nanocomposite free of chemical waste and impurities that may support cross-chemical effects [57–60]. Indeed, plant-derived green synthesis is not only preferred over chemical routes, but also comes first in the hierarchy of biological synthetic approaches, as it eliminates cost/time/effort exhaustion in preserving bio-cultures for large scale production [61]. In total, suitable raw materials were integrated by a novel, green, impurity-free synthesis approach, for a multi-functional hybrid with complementary advanced features.

**Fig 2:**
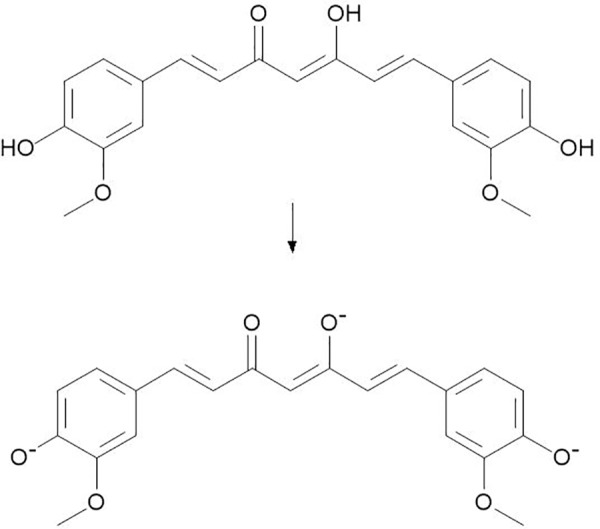
The proposed reduction sites action of CR. The O^-^ moieties formed at alkaline pH are assumed to reduce Au^+^ ions to AuNPs [63].

The expected mechanism behind CAG synthesis is explained initially by the opposite charge attraction between oxygen functionalities of GO and Au^3+^ ions in solution [62], followed by reduction in-place to form AuNPs via CR action [62]. The alkaline pH together with the suitable molar ratio were applied based on previous optimization studies to ensure CR peak reduction [63]. The resulting CR anions are expected to be the ingredient responsible for the reduction. This mechanism is thought to progress through six stages, as explained by Sindhu et.al (2014), using CR in nano-synthesis. The stages extend for 4 hours and include deprotonation of CR, gold ion reduction, nucleation, growth, cleavage, and finally, maturation [63]. AuNPs is expected to be stabilized by ionized CR molecules on the CAG surface. Fig 2 represents the possible reduction sites of Au ions by CR as concluded and reported previously [63]. Characterization results, as explained below, collectively confirm this mechanism and assure successful utilization of CR as a powerful reducing and capping agent.

### Morphological and structural features

**Fig 3:**
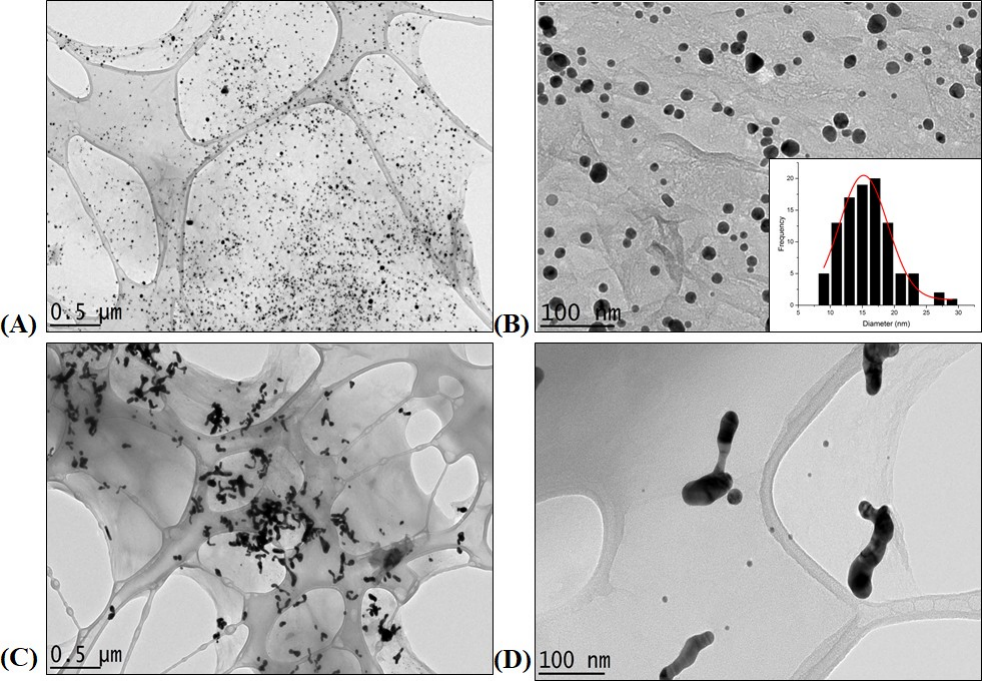
TEM images of CAG vs. rGO-AuNP nanocomposites. (A) CAG composite at 0.5 μm scale, (B) CAG at 100 nm scale with an inset of size distribution histogram. (C) rGO-AuNPs prepared using sodium citrate at 0.5 μm scale, (D) rGO-AuNPs at 100 nm scale.

Transmission electron microscopy (TEM) images (Fig 3) clearly show the successful synthesis of nanocomposite rGO-AuNPs using CR to yield CAG. This synthetic route was compared to traditional *in-situ* reduction synthesis of an rGO-AuNP composite using sodium citrate [43]. Both images displayed the typical sheet-like morphology of graphene; however, the morphology of AuNPs anchored to the surface differs significantly. As shown, AuNPs are well-dispersed, with nearly uniform and homogenous spherical shape and size (15.62 ± 4.04 nm), using CR as the reducing and capping agent in the CAG composite. On the other hand, citrate produced aggregated AuNPs on the rGO surface. This finding is in agreement with previous studies that report aggregated AuNP formation on the rGO surface because of weak citrate function as a capping agent [43]. The mechanism of AuNP aggregation was explained by Chuang et.al (2014), as free-standing AuNPs formed in solution displace citrate ions, which weakly cap anchored AuNPs on the rGO surface. This leads to the formation of chain-like aggregated AuNPs [43]. The TEM results indicated that CR not only reduces gold salt to AuNPs, but also provides a strong capping and stabilizing action to the formed AuNPs on the rGO surface, preventing their aggregation without the need to add additional chemicals as used previously [43]. The remaining AuNPs in solution can be easily removed by washing and purification steps followed in the synthesis protocol [43].

**Fig 4:**
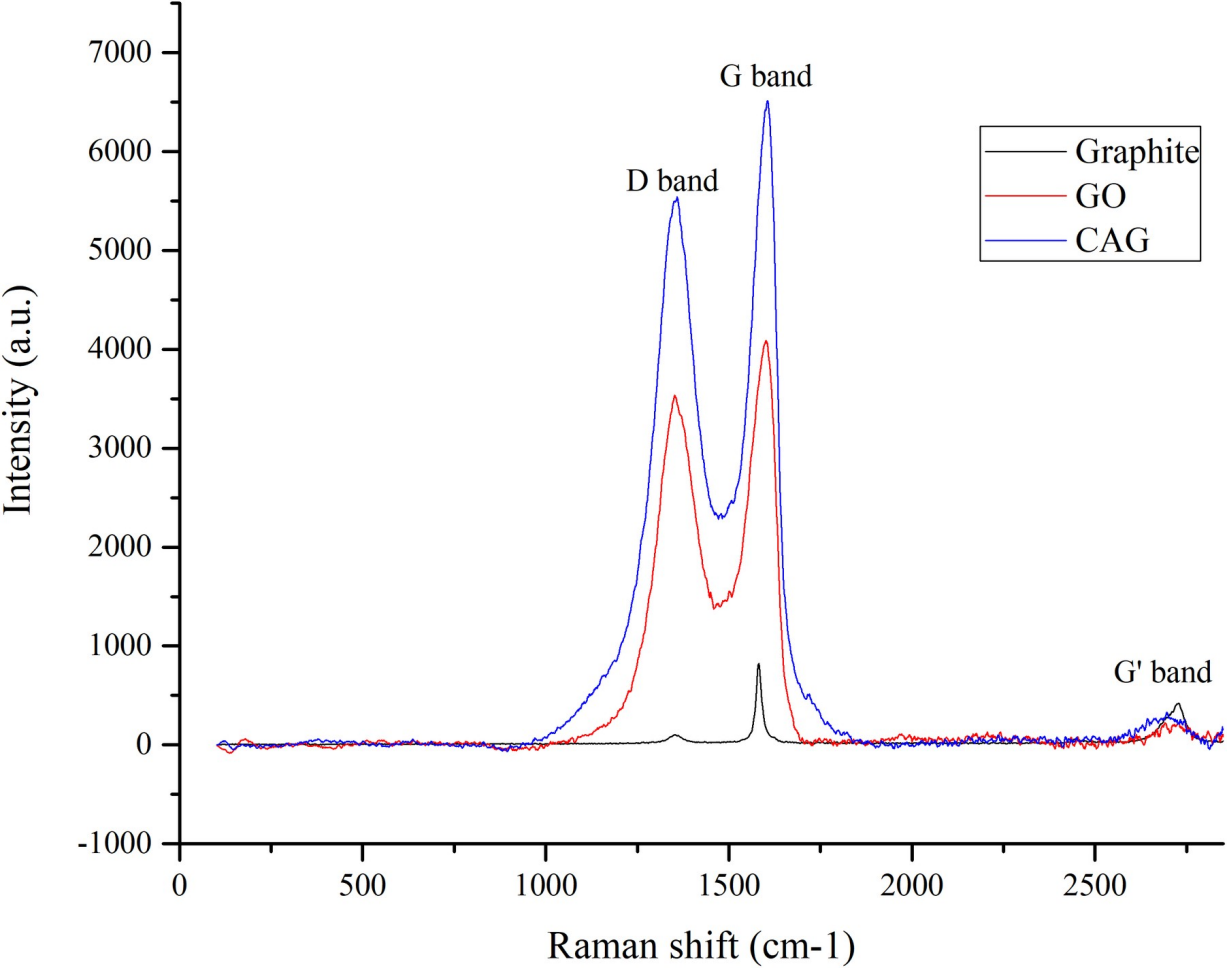
Raman spectra of synthesized nanocomposite CAG, GO and graphite.

Raman spectroscopy is a potent technique to reveal graphene structural changes upon functionalization [64–67]. In general, graphene-based composites exhibit characteristic G bands (1580 cm^-1^) indicative of sp^2^-hybridized carbon atoms stretch, and G’ bands (2500–2800 cm^-1^) [65, 66]. A Raman spectrum was recorded for CAG nanocomposite in comparison with raw materials used in the synthesis of graphite and GO, as shown in Fig 4. Both G and G’ bands appeared in all tested composites, which assures a preserved graphitic template after functionalization reaction with AuNPs and CR. On the other hand, a disorder-induced D band in GO and CAG nanocomposite spectra refers to sp^3^-hybridized carbon systems, resulting from the functionalization out of raw pure graphite [66]. The novel nanocomposite CAG spectrum revealed upshift in bands position (G and D bands at 1605, and 1358 cm^-1^), compared to graphite (1581, 1352 cm^-1^) and GO (1600, and 1351 cm^-1^), respectively. This further confirms AuNPs interaction and doping on graphene surface [68, 69]. Additionally, the enhanced intensity values observed in CAG spectrum reflects surface enhanced Raman resonance (SERS) effect, as reported previously for AuNPs-functionalized-graphene composites [68, 70].

The intensity ratio I_D_/I_G_ is commonly reported to assess the sp^2^ domain extent in graphene composites [65]. The starting material GO scored a ratio of 0.86, while it reached 0.85 for CAG nanocomposite. The slight decrease in ratio marks partial reduction of GO to rGO by CR action [43, 65, 71], which is consistent with the reducing nature of CR, and its interaction with some oxygen functionalities of GO [43, 64].

**Fig 5:**
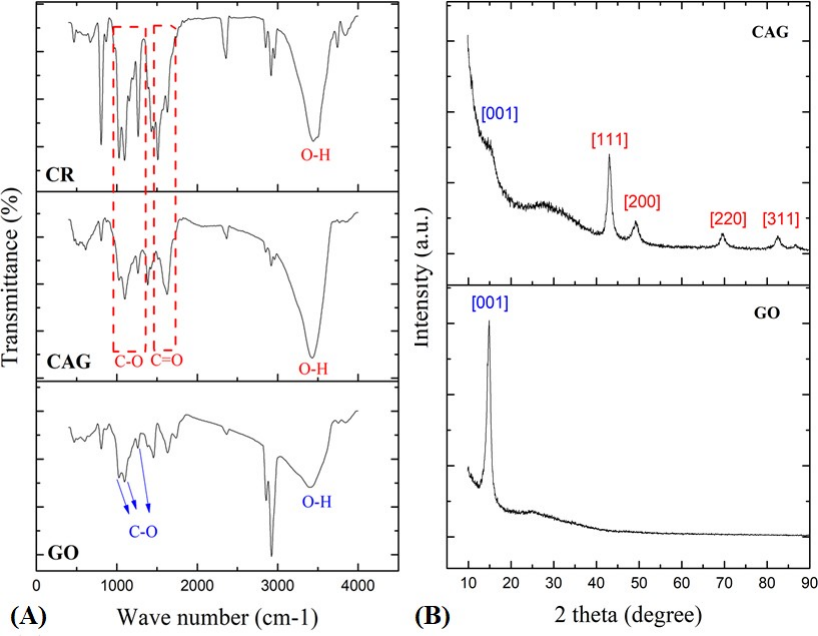
FTIR and XRD analysis. (A) FT-IR spectra of GO, CAG, and CR. (B) XRD analysis of GO and CAG.

The presence of the functional group, including CR moieties attached to the CAG surface, was studied by Fourier transform infrared spectroscopy (FT-IR). Fig 5A presents the FT-IR spectra of the CAG composite compared to GO and CR. The GO spectrum shows multiple peaks that correspond to different O_2_-functional groups. The most intense peaks at 2857 and 2923 cm^-1^ represent methyl C-H bond for graphene structure [72]. Other prominent peaks at 1027 cm^-1^, 1096 cm^-1^, and 1260 cm^-1^ reflect absorbance intensities of carbon-oxygen (C-O) bonds [65]. However, some of these peaks disappeared or appeared with only weak absorbance in the CAG composite, which indicates reduction to rGO by curcumin action. Herein, CAG exhibited a reduced graphene pattern as cited in previous studies, whereby O_2_ functional groups appeared at 1090 cm^-1^ (C-O stretch) but with low intensity [73, 74], as well as peaks at 1576 cm^-1^ corresponding to C = C [64]. However, additional fingerprint peaks were observed at 1026 cm^-1^, 1099 cm^-1^, and 1263 cm^-1^, indicating curcumin presence in its intact form with bonds of (C-O) and carbonyl (C = O) at 1625 cm^-1^ [63–65]. By comparison with a pure curcumin spectrum, the multiple peaks at a range of 1028–1510 cm^-1^ appeared as well in the CAG composite, which in turn confirms the successful interaction of CR with graphene and gold [63, 64]. The bands observed at 1265 cm^-1^, 1384 cm^-1^, and 969 cm^-1^ in the CR spectrum reflect hydroxyl stretches of phenolic and enolic groups, respectively. These bands appeared with only low intensities in CAG, which may explain the interaction sites of CR to Au on the rGO surface [63], as explained and illustrated earlier in text (Fig 2).

To confirm the successful formation and attachment of AuNPs to the rGO surface, X-ray diffraction (XRD) analysis was used, which further helps to explore the crystalline structure of the composite. Fig 5B shows XRD patterns of CAG in addition to GO, which served as a precursor for synthesis. As shown, GO had a characteristic XRD pattern with an intense peak at 10°C (2θ), which corresponds to the crystalline orientation of (001) and d-interlayer spacing of ~0.88 nm due to oxygen-functionalized groups exfoliating carbon sheets [42, 64, 75, 76]. In contrast, the 10°C peak was significantly decreased in intensity in the CAG diffractogram, indicating reduction of oxygen-groups into rGO and restoring the graphene sp^2^ network, giving a characteristic feature of a broad peak at 22.12°C [64, 74, 77]. Formation of AuNPs can be determined by the presence of 2θ peaks at around 38°C, 44.3°C, 64.7°C, and 77.7°C, corresponding to the formation of pure metallic gold with crystal faces (111), (200), (220), and (311), respectively [74, 77]. These peaks were observed in the CAG nanocomposite, confirming the anchoring of AuNPs on the rGO surface through functionalization [74]. Collectively, it has been proven that CR successfully played dual roles in CAG synthesis, reducing both gold salt into AuNPs, and also GO into rGO sheets.

**Fig 6:**
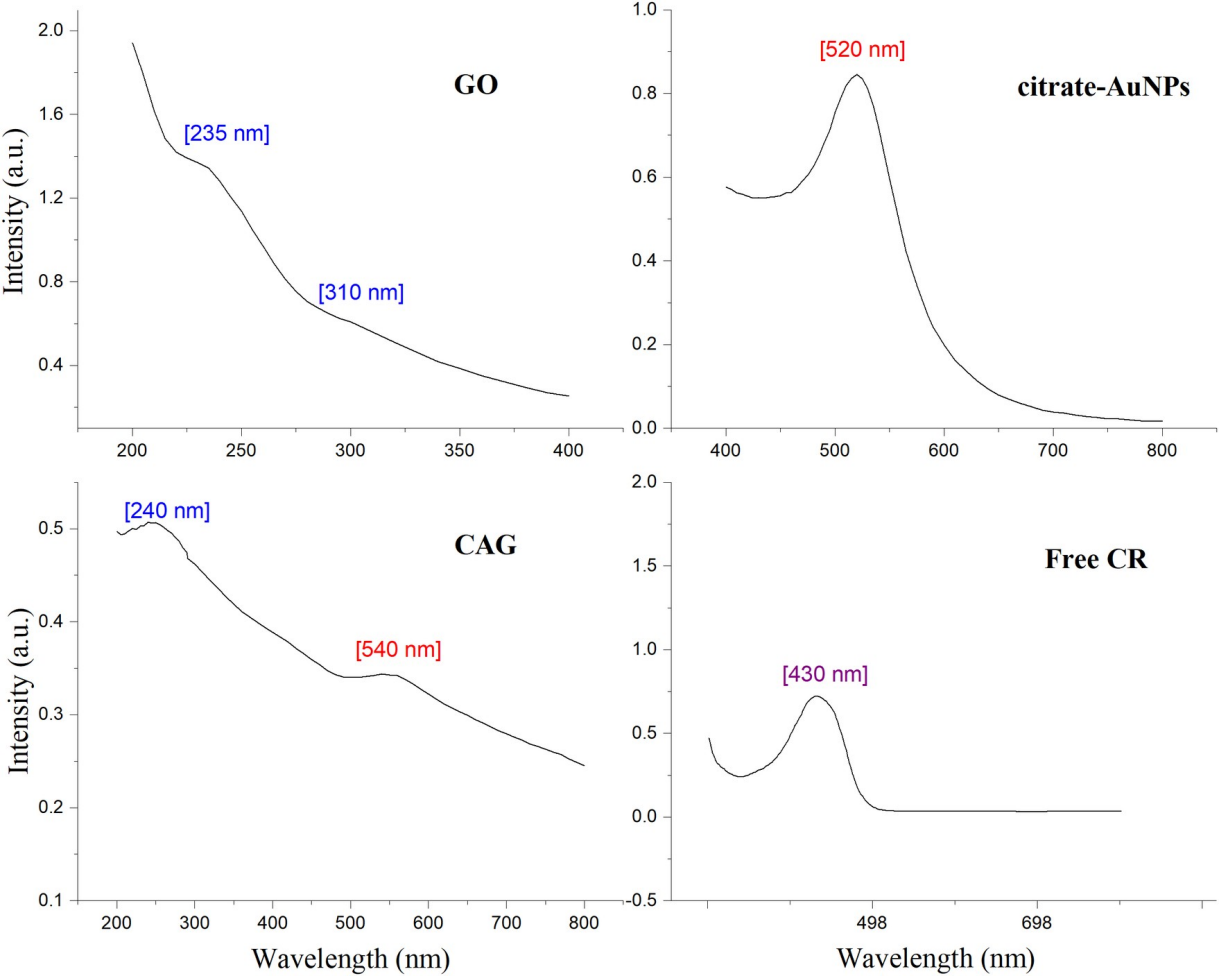
UV-Vis absorption spectra. Panel of GO, citrate-AuNPs, CAG, and CR.

Additional confirmatory results of CAG structural features discussed earlier can be obtained through UV-Vis absorption analysis (Fig 6). Typically, GO exhibits an absorption peak at around 235 nm with a weak shoulder at around 310 nm due to C = C and C = O bonds, respectively [42, 64, 78]. On the other hand, AuNPs shows a dominant absorption peak at 520 nm [44, 79]. The CAG nanocomposite UV-Vis pattern revealed a shifted peak of GO into ~255 nm absorption wavelength, which indicates the reduction process of GO into rGO by CR action [64, 65]. Formation of AuNPs on the CAG surface is confirmed by the presence of a 540 nm absorption peak [63]. The peak shift from 520 to 540 nm is reported as a confirming indicator for the deposition of Au on graphene sheets [74]. Furthermore, a CR prominent absorption peak observed at 430 nm disappeared upon mixing with the CAG composite, denoting chromophore interaction with the rGO surface [64].

**Fig 7:**
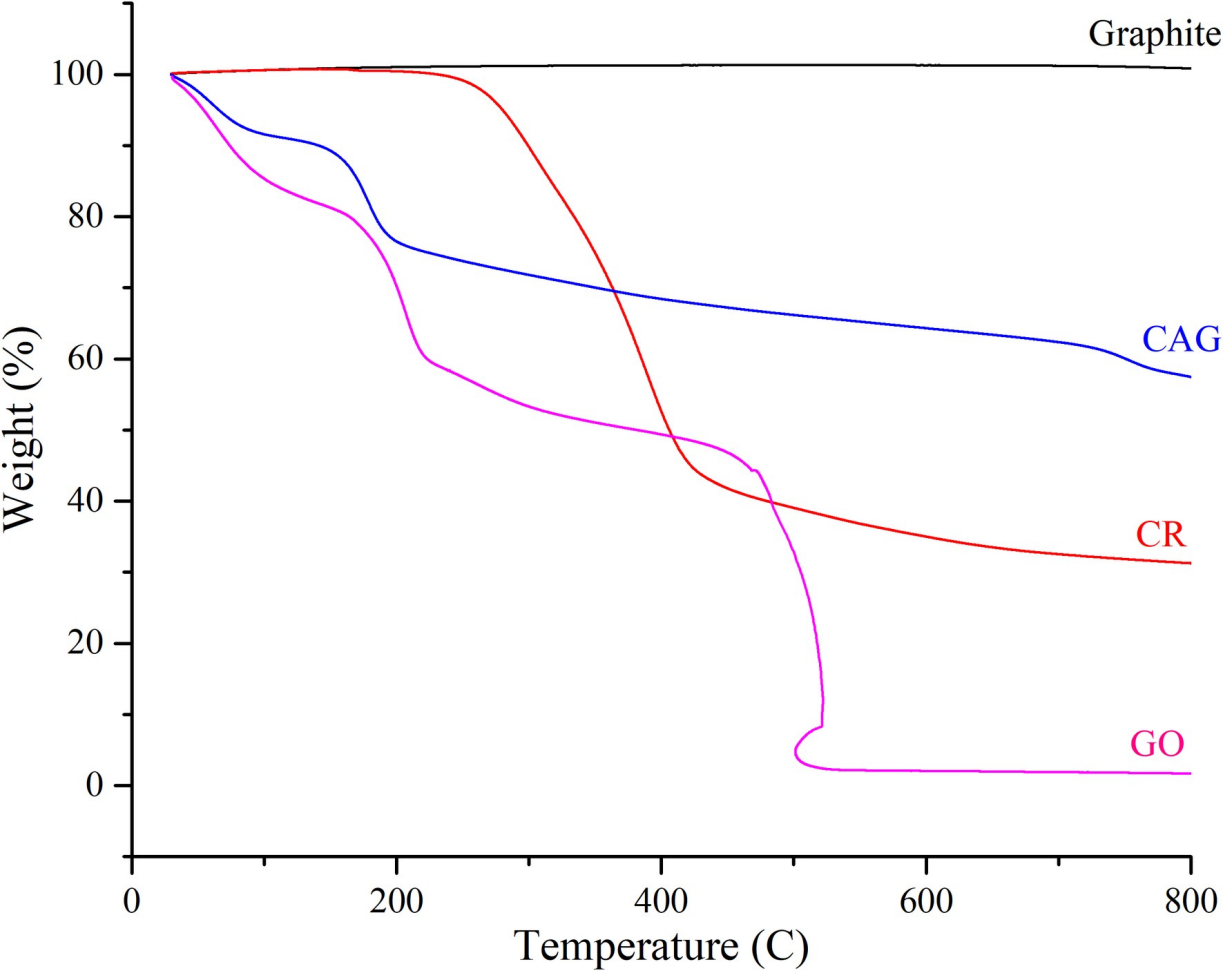
TGA curve trends. TGA curves for CAG in comparison to graphite, CR, and GO.

Functionalization on the carbonaceous nanocomposite surface can be also examined by thermogravimetric analysis (TGA) [64]. Fig 7 shows TGA curves under nitrogen gas flow for CAG in comparison to graphite, GO, and CR. Raw graphite demonstrated high thermal stability with no weight loss over the entire temperature range [64, 80]. On the other hand, functionalized graphene composites in this study showed multi-stage decomposition patterns. GO thermal degradation can be classified into 3 stages, the first weight loss of ~19.5% occurred at temperature 159°C, attributed to water evaporation. The second weight loss of 52.5% occurred at temperature range 159–440°C, related to the removal of oxygen-functional groups. The final stage at 440–528°C left residual weight of only 2.3%, due to combustion and formation of carbon dioxide [64, 81]. The commercial curcumin TGA trend exhibited the signature thermal stability curve [64]. The two mass losses occurred at 240°C and 435°C, reflecting the removal of oxygen groups and combustion, respectively. The novel nanocomposite CAG displayed a similar TGA curve to that of GO, but with a lower weight loss rate, confirming the reduced nature of graphene in the nanocomposite, in agreement with rGO TGA trends seen in literature [80–82]. Additionally, CAG exhibited a multi-staged curve as seen in both GO and CR. A modest weight loss of 9.7% occurred at 136°C, which can be attributed to water removal. Followed by a steep drop in weight percentage until 200°C with total loss of 23.5%, which is thought to be related to the removal of oxygen functional groups of CR attached to the composite surface, where a similar trend was observed in previous studies utilizing CR in nano-synthesis [64]. The final gradual weight loss began at over 200°C, with residual materials of 58.7% and 41.3% weight loss. Thus, the TGA trend of CAG nanocomposite has confirmed the reduced nature of GO [80–82] parallel with the successful functionalization of CR on the surface, which collectively increased the thermal stability compared to GO [64, 65].

**Fig 8:**
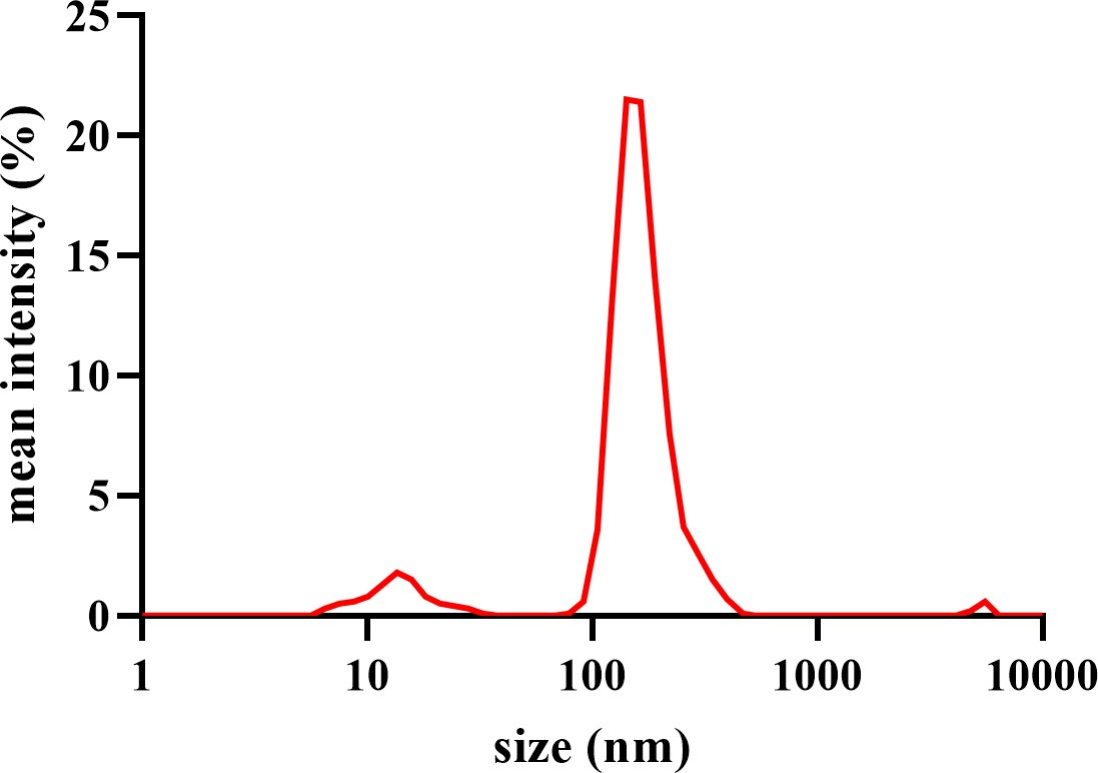
Dynamic light scattering. Particle size distribution of CAG nanocomposite dispersion in RPMI-1640 medium.

Aiming for biological, anti-cancer applications require understanding of nanocomposite’s bio-physical characteristics [83]. Dynamic light scattering enables measuring particles’ hydrodynamic size distribution in biological medium, which is vital to assess dispersion stability [56, 83]. Fig 8 displays the average hydrodynamic diameter of CAG in RPMI-1640 medium (171.3 ± 30.3 nm). The presence of low-intensity peaks at different size regions represents a common finding reported in previous studies working on GBCs, due to sheet-like structures effect on DLS system that is best suited for circular particles [84–87].

### Anti-oxidant activity

**Fig 9:**
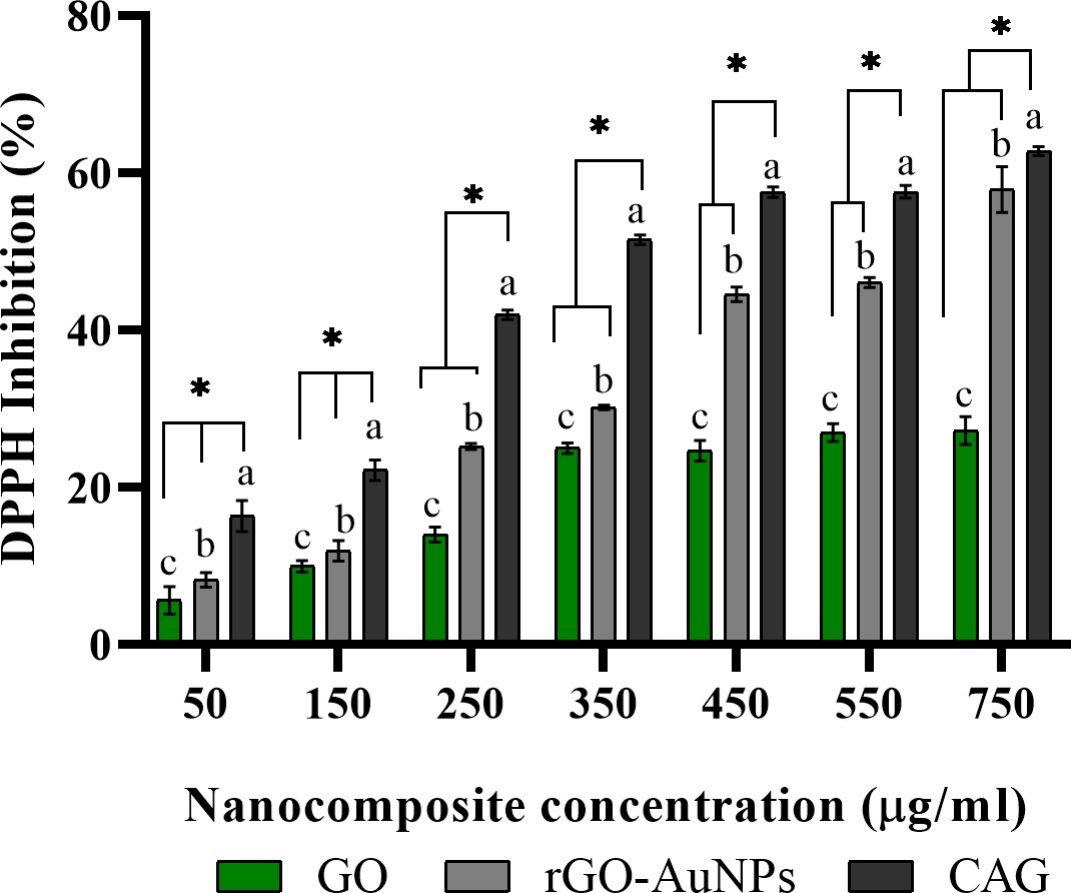
DPPH antioxidant assay. CAG antioxidant activity compared to raw material GO, and citrate synthesized rGO-AuNPs nanocomposite. Results expressed as mean from triplicate analysis ± SEM. ^a, b^ and ^c^ indicates a significant increase in DPPH inhibition of respective treatments (CAG, rGO-AuNPs, and GO, respectively) compared to no treatment control (p<0.05). * Significant difference between treatment groups at respective concentration (p<0.05). Statistical analysis was performed using factorial ANOVA tests, SPSS software.

CR is well known to exhibit a strong antioxidant activity, as evidenced by previous *in vitro* and *in vivo* studies [35, 88–92], which correlate with its anticancer and chemo-preventive actions [93]. Thus, it is anticipated that hybridization of CR with rGO-AuNP nanocomposite will yield superior anti-oxidant potential. Free radical inhibition assessment is currently well-established procedure for nano-synthesized systems intended for biological applications, providing a critical preliminary indication for composite function and activity [94–96]. The nanocomposite CAG was investigated for anti-oxidant properties using 2,2-diphenyl-1-picrylhydrazyl (DPPH) free radical scavenging assay. Furthermore, the raw material used in synthesis–GO, and the traditionally fabricated citrate rGO-AuNPs composite were analyzed as well, for the sake of obtaining a better understanding of the CR effect added to the hybrid system. The antioxidant activity exerted by CAG on DPPH free radicals follows a concentration-dependent manner, as displayed in Fig 9, with obviously amplified inhibition rates compared to raw GO and citrate-rGO-AuNPs at all tested concentrations. The IC_50_ values were determined as 324.1 ± 1.8, 612.1 ± 10.1, and above 750 μg/mL (mean ± SEM) for composites CAG < citrate-rGO-AuNPs < GO, respectively. Standard antioxidant ascorbic acid as positive control along with free curcumin validated the assay results obtained (IC_50_ = 4.53 ± 0.05 and 6.84 ± 0.15%, respectively [97, 98] (S1 Fig). The observed trend suggests that CR incorporation in the nanocomposite was key for tremendous anti-oxidant enhancement, which effectively reduced the IC_50_ value approximately by half, compared to that of the rGO-AuNP traditional composite.

The proposed mechanism by which the nanocomposite CAG acts on DPPH is thought to depend on CR moiety, and to a lesser extent on AuNPs, rather than rGO. In the current experimental results, GO as a raw material was relatively inactive, with the highest concentration inhibiting less than 30% of the free radicals. The addition of AuNPs through sodium citrate conventional synthesis method enhanced the anti-oxidant activity. However, remarkable inhibition rates were achieved using CR green hybridization of GO and AuNPs. These findings correspond with existing literature demonstrating inactivity of the graphene-based family (graphene, rGO, and GO) towards DPPH free radicals, due to poor H-donation ability [99]. Furthermore, previous studies have indicated low antioxidant activity of rGO, even when prepared using green reducing agents. Spinach and clove extract were used as green reductants to prepare reduced-GO, and both scored high IC_50_ values of 1590 and 1337 μg/mL, respectively [100, 101]. On the other hand, the majority of reports confirm that AuNPs has the ability to scavenge the DPPH free radical, in consistent with our experimental result (S1 Fig) [46, 102–105]. This scavenging power is attributed to the DPPH adsorption and complex formation on AuNPs surface, which indicates the displacement of some citrate ions when sodium citrate is used in reduction [104]. In the newly synthesized nanocomposite CAG herein, it is expected that both CR and AuNPs exert radical inhibition in a multi-potent model, where CR plays the major role by its high reducing power and phenolic content, which facilitate H-donation from phenolic–OH groups to DPPH reagent [89].

Total phenolic content was determined in the CAG nanocomposite as it provides a measurement for the active functional CR on surface. The quantitative TPC was derived from a standard curve prepared from ethanolic CR solution [46], and was calculated as 7.45 ± 0.13% (w/w) (mean ± SEM from triplicate analysis) for the synthesized CAG nanocomposite. The overall antioxidant results of the CAG nanocomposite hold great promise as an efficient nano-antioxidant system with multi-potent moieties valuable for various biological and anti-cancer applications.

### Colon cancer morphology, cytotoxicity and selectivity index

The strong well-known anti-oxidant and anti-cancer properties of CR have prompted the application of CAG in cancer studies, along with encouraging results from TPC and DPPH anti-oxidant assays. The evaluation of CAG as an anti-cancer agent was studied using an *in vitro* colon cancer model through cellular morphologic changes and viability measurements. Optical phase-contrast microscopy provides an essential tool to visualize morphological changes which serve as crucial indication for treatment efficacy in inducing cell injury and death [106–108]. Viability assay, on the other hand, determines intracellular physiological capabilities and the metabolic states of treated cells. This accurate, reproducible approach constitutes the first stage of the recommended nanomaterial toxicology evaluation [109, 110]. The well-established water soluble tetrazolium assay (WST-8) was selected for its accuracy and superior detection sensitivity, which surpasses other tetrazolium reduction dyes used in cytotoxicity [111]. Its negative charge enables water soluble tetrazolium dye to be used in this experiment without interference with graphene particles, as indicated previously for MTT [49].

Colon cancer was adopted in this work to elucidate CAG effectiveness as a cancer cytotoxic agent and its selectivity indices, due to the well-suited CR and graphene properties. CR is proven to be effective against almost all cancer types *in vitro*, with colon cancer exhibiting some of the lowest IC_50_ values recorded, which indicate a high susceptibility concept. Studies have shown that deep colorectal tissues gain only around 5% of orally ingested CR [35], which is in agreement with low CR absorption and cellular uptake, and the efflux mechanisms reported from cancer cells [112–114]. These limitations create motives to design a nano-based CR hybrid that facilitates deeper penetration and delivery into colon tissues. To this end, graphene constitutes a promising platform for insoluble drug delivery [115]. Additionally, in a recent work by Lin et al. (2018), graphene oxide itself induced higher cytotoxicity towards colon cancer cells, among different panels of cancer types tested [116]. This remarkably points to a potential synergistic effect exerted by graphene and the loaded drug against colon cancer. Such approach can be termed ‘design-for-purpose’, which is believed to hold high potential and efficiency in applications [117–119].

**Fig 10:**
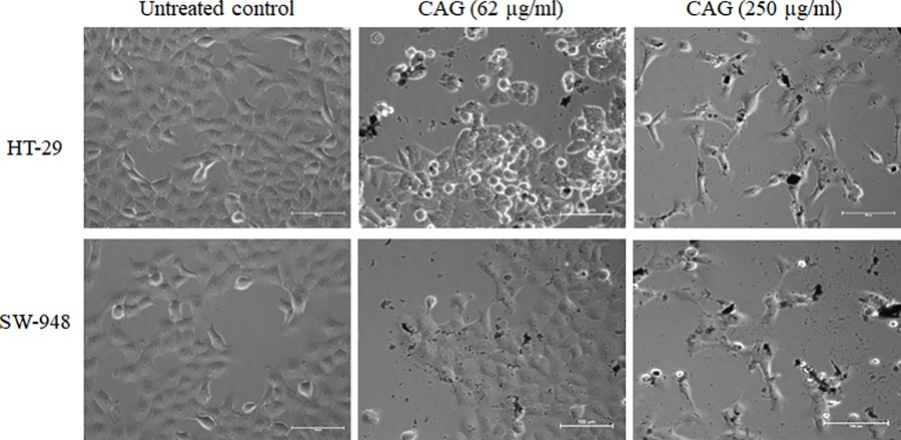
Optical phase-contrast microscopy images. Untreated control cells (up; HT-29, bottom; SW-948) compared to CAG nanocomposite treatment at low and high concentrations.

Two human colon cancer cell lines were tested in this study representing different subtypes, molecular platforms, and prognosis rates: HT-29 (colon adenocarcinoma) with general good prognosis, and SW948 (Duke’s C colorectal carcinoma) with poor prognosis [120]. Fig 10 demonstrates cellular changes as viewed by optical microscope upon CAG treatment. Both cell lines showed distinct size and morphology alteration compared to the untreated control. Cellular shrinkage and proliferation inhibition were observed in CAG treatment groups, which increased with higher CAG concentration, suggesting a dose-dependent trend [121, 122]. Furthermore, morphological changes were studied with starting raw material GO, and traditional citrate-synthesized nanocomposite rGO-AuNPs, as a comparative approach (S2 Fig). GO treated cells showed severe toxicity even at low concentration, which is reported previously reflecting oxygen functionalities reactivity, that may affect GO biocompatibility in applications [115, 123]. On the other hand, rGO-AuNPs showed mild morphological changes that is less adverse than CAG, proposing the possible CR effect in CAG.

**Fig 11:**
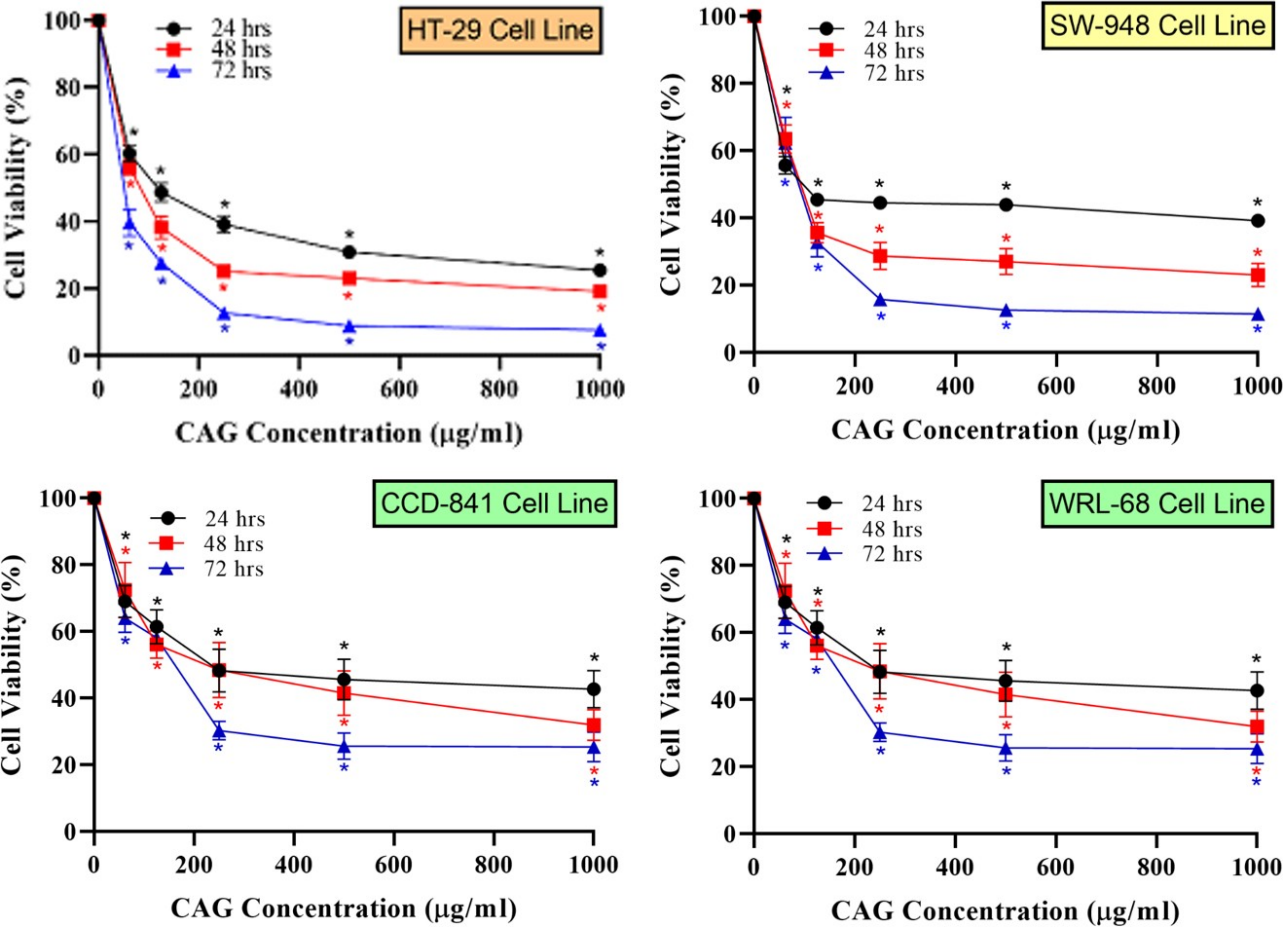
*In vitro* viability results after CAG treatment. Percentage viability of colon cancer cells (HT-29 and SW948 cell lines), normal colon cells (CCD841), and normal liver cells (WRL-68), upon exposure to CAG nanocomposite at different concentrations (62.5–1000 μg/mL), measured at three time points using WST-8 assay. Tests were performed and results were means from triplicate analysis. * Significant decrease (p<0.05) in viability percentage compared to untreated control, as analyzed by factorial ANOVA test, SPSS software.

In support of the previous observations, WST-8 viability assay was conducted using various concentrations and time points. Results as presented in Fig 11, show both time- and concentration-dependent colon cancer cells inhibition in response to CAG treatment. The IC_50_ values, displayed in Table 1, demonstrate the anti-cancer effect of CAG, inhibiting 50% of cell populations at concentrations of approximately 100 μg/mL or below. These results confirm potent anti-cancer activity, which represents one of the objectives of current work. Too often, *in vitro* nano-toxicology studies would stop at this point, concluding the delivery of nanomaterial with anti-cancer properties [41]. However, a realistic optimal nano-medicine demands a broader strategy, accounting for proof of selectivity and normal cell biocompatibility, uncovering the whole picture and providing better perception of the nanomaterial under study. For that reason, parallel assessment of CAG nanocomposite on a benign cell line is conducted to accurately probe the safety profile [41]. The normal colon cell line (CCD-841) demonstrated low inhibition rates at all applied concentrations and time points. The high IC_50_ values (S2 Table) recorded for CCD-841 had proven the safety of CAG on normal colon tissue with SI exceeding 2.0 –the accepted threshold for selectivity of compounds [50]–at all tested time points.

**Table 1:** IC_50_ values and selectivity index (SI) of cancer cells treated with nanocomposites.

	HT-29 (IC_50_ ± SEM μg/mL), SI			SW-948 (IC_50_ ± SEM μg/mL), SI		
	24 hrs.	48 hrs.	72 hrs.	24 hrs.	48 hrs.	72 hrs.
**CAG**	(107.8 ± 8.9), 2.1	(91.7 ± 8.0), 2.3	(59.1 ± 8.0), 2.7	(100.1 ± 8.3), 2.3	(94.0 ± 6.5), 2.2	(79.7 ± 14.2), 2.0
**rGO-AuNPs**	(210.8±11.1), 1.1	(149.9±17.4), 1.1	(115.3±4.5), 1.3	(151.9±16.7), 1.5	(130.6±6.1), 1.2	(101.7±16.4), 1.5
**GO**	(95.7 ± 9.5), 1.0	(56.3 ± 1.6), 1.2	(46.1 ± 0.5), 0.8	(72.4 ± 1.3), 1.3	(71.7 ± 2.6), 0.9	(59.6 ± 1.0), 0.6

As a further verification of the integrated hybrid suggested at the introduction, the anti-cancer activity and SI were measured for GO and rGO-AuNPs as well, to confirm the superiority of green synthesis and CR functionality for drugs with cancer applications. Results were expressed as IC_50_ values and respective selectivity indices, as shown in Table 1. GO caused severe viability inhibition not only to cancer cells but also to normal cell lines, clearly evidenced through the low SI, which is consistent with morphological results explained earlier. The high O_2_-functionality contents in GO can act as electron donors, causing oxidative stress-induced cytotoxicity, among other cellular damaging effects [115, 123]. In response, various strategies were recommended to minimize cytotoxicity while maintaining solubility, which includes the use of rGO instead of GO [123], as well as functionalization with biocompatible AuNPs [11, 24, 115, 124]. These factors can explain the enhanced selectivity indices of rGO-AuNPs and CAG nanocomposites seen in Table 1, reflecting the reduced graphene nature, along with AuNPs functionalization. Nonetheless, the acceptable SI was achieved only when applying CAG, distinctly manifesting the CR role and green synthesis effect with SI percentage enhancement of ≥90% for HT-29 and ≥30% for SW-948 cancer cells, compared to traditional rGO-AuNPs.

**Fig 12:**
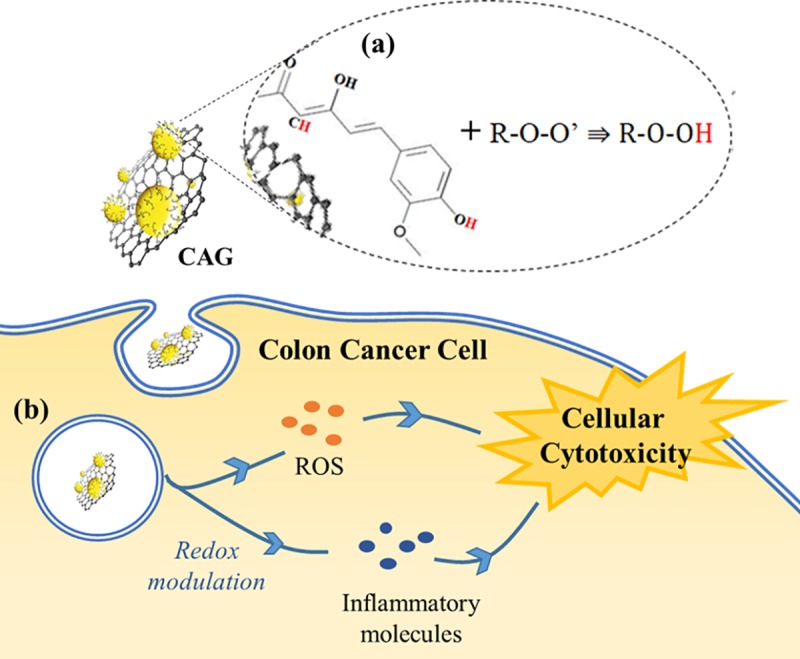
Schematic illustration of the proposed CAG interaction and mechanism of action on cell. (a) Antioxidant activity and free radical inhibition, (b) CAG-cell interaction and proposed subsequent mechanism.

Towards comprehensive selectivity evaluation, CAG was further tested for SI in liver normal cells (WRL-68) as RES organ, standing for a possible model of CAG clearance and deposition, reported in the literature for graphene-based materials tested *in vivo* [39, 40]. We believe that this step will aid in relating to CAG behavior in a biological setting, providing an important glimpse of the suitability of nano-system design and function. Similar to colon tissue, liver normal cells showed low inhibition rates with allowable SI (S3 Table). Collectively, results obtained in this work suggests a possible interactive pathway of cells with CAG nanocomposite, as illustrated in Fig 12. The well-documented graphene-cell interaction and uptake [123] is proposed to facilitate subsequent intracellular activities. The nanohybrid with CR moiety effectively inhibit free radicals, as proven by DPPH assay, which is thought to be the vital key in projecting superior cancer cytotoxicity effects, through counteracting and redox modulation of the built-in high ROS and inflammatory intracellular tumor environment [125]. In fact, redox-modulation cytotoxicity symbolizes the prominent pathway for both CR-based nano-formulations, and nano-toxicology materials in general as reported in previous research [121, 126–131].

## Conclusion

This study presented the novel, green, fabricated nanocomposite CAG and demonstrated its effectiveness as an antioxidant and selective anti-cancer agent. The raw material selection was driven by the individual effective features, leading to simple, one-pot, green incorporation and investment into a superior integrated hybrid system, with maximal use of CR beneficial multi-functional attributes. Characterization results demonstrate a CR functionalized structure with dispersed homogenous AuNPs with no added stabilizer, surpassing a major problem reported previously with traditional chemical reductants. Our data also showed potent anti-oxidant and anti-cancer properties with *in vitro* colon cell lines. The preserved selectivity among various tissue representatives was further proof of reliable *in vitro* nano-medicine holding great promise for further development and assessments. The sum of data reported herein strongly suggests that precise planning for nanomaterial fabrication, green synthesis, and careful selectivity measurements add much to the output nanocomposite designated for biological applications.

## Supporting information

S1 Table Examples of GBCs synthesized in previous studies by green chemistry for cancer applications. (DOCX)

S2 Table IC_50_ values of colon normal cell line CCD-841 treated with nanocomposites at different time points. Measurements obtained through the WST-8 assay. Results were expressed as mean ± SEM (μg/mL) from triplicate analysis.
(DOCX)

S3 Table IC_50_ values of the liver normal cell line WRL-68 treated with the CAG nanocomposite at different time points. Measurements obtained through the WST-8 assay. Results were expressed as mean ± SEM (μg/mL) from triplicate analysis.
(DOCX)

S1 Fig DPPH free radical inhibition assay. (a) Standard ascorbic acid, (b) free CR, (c) sodium citrate-gold nanoparticles (AuNPs). Results were expressed as mean ± SEM (μg/mL) from triplicate analysis.
(TIF)

S2 Fig Optical microscopy images of GO and rGO-AuNPs treated cancer cell lines. (TIF)
