# Supplementary material for: Hybrid nanocomposite curcumin-capped gold nanoparticle-reduced graphene oxide: Anti-oxidant potency and selective cancer cytotoxicity
Source: PLoS One. 2019 May 14;14(5):e0216725. doi: 10.1371/journal.pone.0216725 (PMC6516671; doi:10.1371/journal.pone.0216725)
Supplement: S1 Table — (DOCX) [file pone.0216725.s001.docx]

S1 Table: Examples of GBCs synthesized in previous studies by green chemistry for cancer applications.

| GBCs | Green synthesis approach/agent | Human cancer model | Human normal cells | SI | reference |
| --- | --- | --- | --- | --- | --- |
| CAG (Current study) | curcumin | *In vitro* colon cancer HT29 and SW948. | *In vitro* normal colon CCD841 and normal liver cells WRL-68. | Above 2.0 at all tested time points. | Current study |
| CR-rGO | curcumin | Breast cancer MDA and SKB | Normal mice fibroblast L929 | Not determined. Reported similar effects  On cancer and normal cells | [[1](#_ENREF_1)] |
| Biologically-reduced GO by *Bacillus marisflavi* biomass | Bacteria species. | MCF7 breast cancer cell line | - | - | [[2](#_ENREF_2)] |
| *Citrullus colocynthis* poly-phenol-stabilized reduced graphene oxide | leaf extract of *Citrullus colocynthis* | Prostate cancer DU145 | - | - | [[3](#_ENREF_3)] |
| Pulicaria glutinosa extract (PGE) for silver NPS-decorated GN | *Pulicaria glutinosa* extract (PGE) | *In vitro* cell lines: MCF-7 (breast), A549 (lung), Hela (cervical), DU-145 (prostate) and HepG2 (liver) | - | - | [[4](#_ENREF_4)] |
| Doxorubicin/gelatin functionalized graphenenanosheets (gelatin–GNS) | Gelatin as reducing agent | MCF7 breast cancer | - | - | [[5](#_ENREF_5)] |
| Vit C-GO and GO-AgNPs | Vitamin C | Lung cancer A549 | - | - | [[6](#_ENREF_6)] |
| Resveratrol-rGO | resveratrol | Ovarian cancer A2780 | -  - | - | [[7](#_ENREF_7)] |
| DOX-Tea phenols-rGO | Tea polyphenol | Cell line ACC2 | Normal mice-derived cell line L929 | Not determined | [[8](#_ENREF_8)] |

References:

1. Hatamie S, Akhavan O, Sadrnezhaad SK, Ahadian MM, Shirolkar MM, Wang HQ. Curcumin-reduced graphene oxide sheets and their effects on human breast cancer cells. Materials Science and Engineering: C. 2015;55:482-9.

2. Gurunathan S, Han JW, Eppakayala V, Kim J-H. Green synthesis of graphene and its cytotoxic effects in human breast cancer cells. International journal of nanomedicine. 2013;8:1015.

3. Zhu X, Xu X, Liu F, Jin J, Liu L, Zhi Y, et al. Green synthesis of graphene nanosheets and their in vitro cytotoxicity against human prostate cancer (DU 145) cell lines. Nanomaterials and Nanotechnology. 2017;7:1847980417702794.

4. Khan M, Khan M, Al-Marri AH, Al-Warthan A, Alkhathlan HZ, Siddiqui MRH, et al. Apoptosis inducing ability of silver decorated highly reduced graphene oxide nanocomposites in A549 lung cancer. International journal of nanomedicine. 2016;11:873.

5. Liu K, Zhang J-J, Cheng F-F, Zheng T-T, Wang C, Zhu J-J. Green and facile synthesis of highly biocompatible graphene nanosheets and its application for cellular imaging and drug delivery. Journal of Materials Chemistry. 2011;21(32):12034-40.

6. Kavinkumar T, Varunkumar K, Ravikumar V, Manivannan S. Anticancer activity of graphene oxide-reduced graphene oxide-silver nanoparticle composites. Journal of colloid and interface science. 2017;505:1125-33.

7. Gurunathan S, Han JW, Kim ES, Park JH, Kim J-H. Reduction of graphene oxide by resveratrol: a novel and simple biological method for the synthesis of an effective anticancer nanotherapeutic molecule. International journal of nanomedicine. 2015;10:2951.

8. Wang X, Hao L, Zhang C, Chen J, Zhang P. High efficient anti-cancer drug delivery systems using tea polyphenols reduced and functionalized graphene oxide. Journal of biomaterials applications. 2017;31(8):1108-22.
