# Supplementary material for: Hybrid nanocomposite curcumin-capped gold nanoparticle-reduced graphene oxide: Anti-oxidant potency and selective cancer cytotoxicity
Source: PLoS One. 2019 May 14;14(5):e0216725. doi: 10.1371/journal.pone.0216725 (PMC6516671; doi:10.1371/journal.pone.0216725)
Supplement: S2 Table — Measurements obtained through the WST-8 assay. Results were expressed as mean ± SEM (μg/mL) from triplicate analysis. (DOCX) [file pone.0216725.s002.docx]

S2 Table. The IC_50_ values of colon normal cell line CCD-841 treated with nanocomposites at different time points. .

| Nanocomposites | Time points | | |
| --- | --- | --- | --- |
|  | 24 hrs | 48 hrs | 72 hrs |
| CAG | 231.8 ± 26.8 | 207.3 ± 35.5 | 159.9 ± 13.4 |
| rGO-AuNPs | 234.1 ± 12.0 | 162.5 ± 1.2 | 155.5 ± 14.3 |
| GO | 100.1 ± 13 | 65.1 ± 3.22 | 39.9 ± 1.5 |

Measurements obtained through WST-8 assay. Results were expressed as mean ± SEM (µg/mL) from triplicate analysis.
