# Supplementary material for: Hybrid nanocomposite curcumin-capped gold nanoparticle-reduced graphene oxide: Anti-oxidant potency and selective cancer cytotoxicity
Source: PLoS One. 2019 May 14;14(5):e0216725. doi: 10.1371/journal.pone.0216725 (PMC6516671; doi:10.1371/journal.pone.0216725)
Supplement: S3 Table — Measurements obtained through the WST-8 assay. Results were expressed as mean ± SEM (μg/mL) from triplicate analysis. (DOCX) [file pone.0216725.s003.docx]

S3 Table. The IC_50_ values of liver normal cell line WRL-68 treated with CAG nanocomposite at different time points.

| Nanocomposites | Time points | | |
| --- | --- | --- | --- |
|  | 24 hrs | 48 hrs | 72 hrs |
| CAG – IC_50_ | 224.7 ± 20.3 | 199.4 ± 6.3 | 190.4 ± 7.4 |
| Respective SI using HT29 | 2.08 | 2.17 | 3.22 |
| Respective SI using SW948 | 2.24 | 2.12 | 2.38 |

Measurements obtained through WST-8 assay. Results were expressed as mean ± SEM (µg/mL) from triplicate analysis.
