# Supplementary figures and images for: Hybrid nanocomposite curcumin-capped gold nanoparticle-reduced graphene oxide: Anti-oxidant potency and selective cancer cytotoxicity
Source: PLoS One. 2019 May 14;14(5):e0216725. doi: 10.1371/journal.pone.0216725 (PMC6516671; doi:10.1371/journal.pone.0216725)

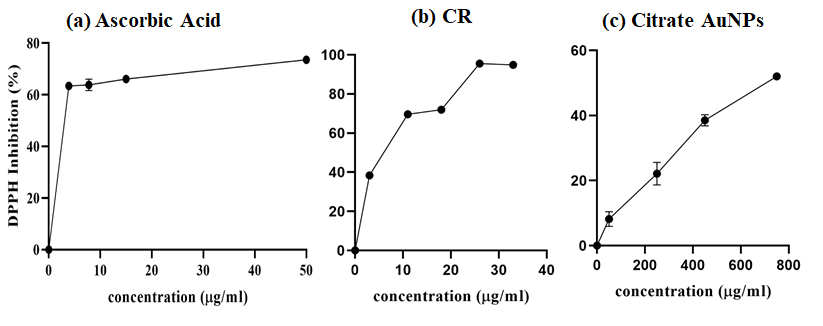

Supplement: S1 Fig — (a) Standard ascorbic acid, (b) free CR, (c) sodium citrate-gold nanoparticles (AuNPs). Results were expressed as mean ± SEM (μg/mL) from triplicate analysis. (TIF) [file pone.0216725.s004.tif]

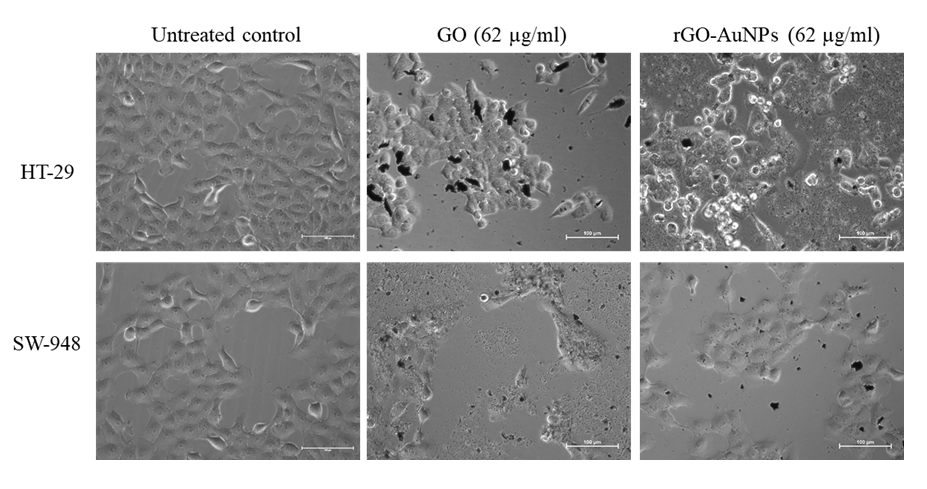

Supplement: S2 Fig — (TIF) [file pone.0216725.s005.tif]
